# Local Versus Global Binarization Techniques After Frangi Filtering for Optical Coherence Tomography Angiography Based Retinal Vessel Density Assessment in Diabetic Retinopathy

**DOI:** 10.3390/diagnostics16060934

**Published:** 2026-03-21

**Authors:** Andrada-Elena Mirescu, Ioana Teodora Tofolean, Sanda Jurja, Florian Balta, Alina Popa-Cherecheanu, Ruxandra Angela Pirvulescu, Gerhard Garhofer, George Balta, Irina-Elena Cristescu, Dan George Deleanu

**Affiliations:** 1 “Ovidius” University of Constanţa, 900470 Constanţa, Romania; 2 “Carol Davila” University of Medicine and Pharmacy, 050747 Bucharest, Romania; florianbalta@gmail.com (F.B.); ruxandrapascu78@gmail.com (R.A.P.); dan-george.deleanu@umfcd.ro (D.G.D.); 3 Clinical Institute of Ophthalmological Emergencies “Prof. Dr. Mircea Olteanu”, 030167 Bucharest, Romania; 4 Retina Clinic, 014142 Bucharest, Romania; cristescu.irina@gmail.com; 5 County Clinical Emergency Hospital of Constanta, 900591 Constanţa, Romania; 6 University Emergency Hospital, 050098 Bucharest, Romania; 7 Department of Clinical Pharmacology, Medical University Vienna, 1090 Vienna, Austria

**Keywords:** diabetic retinopathy, OCTA, vessel density, Frangi filtering, binarization techniques

## Abstract

**Background/Objectives:** Optical coherence tomography angiography (OCTA) enables noninvasive quantitative assessment of the retinal microvasculature and is widely used in diabetic retinopathy (DR). However, OCTA-derived metrics are highly dependent on post-processing techniques, particularly vessel binarization. This study aimed to compare local and global binarization methods applied after Frangi filtering for vessel enhancement in parafoveal vessel density analysis. **Methods:** This cross-sectional study included 69 participants: 17 healthy controls and 52 diabetic patients, classified as the following: no DR (*n* = 14), non-proliferative DR (NPDR, *n* = 18), or proliferative DR (PDR, *n* = 20). All subjects underwent comprehensive ophthalmological examination and OCTA imaging of the superficial capillary plexus using a Topcon OCTA system. Images were processed using a custom MATLAB protocol. Following Frangi filtering, five binarization methods were applied: three local (Phansalkar, local Otsu, adaptive mean) and two global (global mean and global Otsu). Parafoveal vessel density was quantified within the four inner quadrants of the ETDRS grid. **Results:** Statistically significant differences in vessel density were consistently observed between PDR group and both the control and no DR groups across all local binarization methods. Among global methods, only global Otsu thresholding detected a significant difference between PDR and control. The most robust differences were predominantly identified in the nasal and inferior quadrants. **Conclusions:** Local adaptive binarization methods demonstrated superior sensitivity and structural preservation for parafoveal vessel density analysis in DR. Global methods showed limited discriminative capability. These findings support the preferential use of local adaptive techniques for reliable OCTA-based vascular assessment in diabetic retinopathy.

## 1. Introduction

Diabetic eye disease continues to pose an escalating global health challenge, with current estimates predicting a 45% increase in diabetes mellitus worldwide, reaching nearly 852 million affected individuals by 2050 [1,2]. Diabetic retinopathy (DR), a microvascular complication resulting from long-standing hyperglycemia, leads to progressive structural and functional damage within the retina [1]. Clinically, diabetic retinopathy is classified into non-proliferative diabetic retinopathy (NPDR) and proliferative diabetic retinopathy (PDR) stages, the latter characterized by pathologic neovascularization [3,4]. Although diagnosis traditionally begins with a detailed fundoscopic examination, an expanding array of imaging technologies now plays an essential role in screening, grading disease severity, and guiding management strategies [5].

The emergence of optical coherence tomography angiography (OCTA) has transformed the evaluation of retinal microvasculature by enabling high-resolution, noninvasive quantification of blood flow abnormalities [6,7,8,9,10]. Its ability to separately visualize the superficial and deep capillary plexuses (SCP and DCP) provides depth-resolved insight into vascular alterations associated with diabetes [10,11,12]. Numerous clinical studies have used OCTA to characterize diabetic-related microvascular changes within the posterior pole, such as microaneurysms, capillary dropout, vessel density changes, foveal avascular zone (FAZ) area enlargement and neovascularization [10,11,13,14,15]. Importantly, OCTA has also proven valuable in eyes with no or only mild retinopathy, where it can reveal early perfusion deficits that precede clinical findings, thereby enhancing the detection of subclinical disease and offering potential for identifying patients at elevated risk of progression to advanced diabetic retinopathy [5,10].

The Early Treatment Diabetic Retinopathy Study (ETDRS), which developed the ETDRS grid, aimed to assess macular edema relative to the foveal center. It has since that moment become standard and has been integrated into digital imaging systems. The grid was later slightly resized to match the 6 mm macular sampling area. The grid’s center must be aligned with the anatomical center of the macula, the fovea. The map is then divided into nine standardized subfields arranged concentrically around the fovea. At its center lies a 1 mm diameter circular region, surrounded by an inner ring extending to 3 mm and an outer ring extending to 6 mm in diameter. Each ring is split using radial lines into four quadrant subfields: superior, nasal, inferior, and temporal, creating four inner-quarter and four outer-quarter annuluses. Together, the central circle, the four inner subfields, and the four outer subfields form the nine-part ETDRS grid used for regional macular assessment. The map can be used in multiple ways for research purposes such as for retinal thickness analysis or OCTA vessel density measurements by quadrant [16].

Recent advances in image processing have improved the quantitative analysis of OCTA images. Various vessel enhancement and segmentation techniques have been developed to better visualize retinal microvasculature. Among these, the Frangi vesselness filter, a Hessian-based method, enhances tubular structures, thereby highlighting blood vessels while suppressing background noise. This method has been widely used to improve vessel detection and support quantitative analysis in retinal imaging [17].

After vessel enhancement using filters such as the Frangi filter, binarization is performed to convert the vesselness map into a binary image for vessel density quantification in OCTA images. Binarization algorithms can be broadly classified into global methods, which apply a single threshold to the entire image, and local methods, which determine thresholds for different regions based on surrounding pixel characteristics. In this study, five thresholding methods were evaluated: three local methods (Phansalkar, local Otsu, and adaptive mean) and two global methods (global mean and global Otsu) [18].

The aim of the present study was to evaluate retinal vessel density within the parafoveal and inner ETDRS quadrants using different global and local binarization methods applied to OCTA images through a custom-developed MATLAB script, and to determine which processing approach provides the most reliable quantitative assessment in diabetic patients, with or without diabetic retinopathy.

## 2. Materials and Methods

### 2.1. Study Design and Participants

The current study was a cross-sectional analysis conducted on 69 patients, each evaluated at a single time point in our Ophthalmology “Retina” Clinic. The study cohort included 17 healthy volunteers (11 males and 6 females), while the remaining 52 participants were patients with diabetes, stratified into three clinical categories: diabetic patients without diabetic retinopathy (14 patients with no DR—5 male and 9 female), patients with non-proliferative diabetic retinopathy (18 patients with NDPR—10 male and 8 female), and patients with proliferative diabetic retinopathy (20 patients with PDR—15 male and 5 female). The mean age (±standard deviation) was 52.29 ± 13.13 years in the control group, 54.07 ± 21.36 years in the no DR group, 50.27 ± 12.31 years in the NPDR group, and 56.00 ± 13.33 years in the PDR group.

The diabetic cohort included 12 individuals with type I diabetes mellitus (4 no DR, 3 NPDR and 5 PDR), and 40 with type II diabetes mellitus (10 no DR, 15 NPDR and 15 PDR). Among these patients 26 (10 no DR, 7 NPDR, 9 PDR) were insulin-dependent at the time of inclusion. Among the 38 patients diagnosed with NPDR or PDR, 17 had concurrent diabetic maculopathy, 14 had previously undergone panretinal photocoagulation, 19 had received intravitreal anti-vascular endothelial growth factor (anti-VEGF) therapy, and 5 had undergone pars plana vitrectomy. All interventional procedures had been performed more than three months prior to inclusion in the present study.

All assessments were conducted at a single time point. Written informed consent was obtained from each participant. The study received ethical approval from both the Ethics Committee of Ponderas Academic Hospital, Bucharest, Romania (No. 331/18 December 2020) and the Ethics Committee of the “Ovidius” University of Constanta, Constanta, Romania (No. UOC 17057/11 November 2022), and it adhered to the tenets of the Declaration of Helsinki.

### 2.2. Eligibility Criteria

Participants were eligible if they were aged 18 years or older and capable of understanding the ophthalmological procedures involved. Common inclusion criteria across all groups included the following: age ≥18 years, caucasian ethnicity, best-corrected visual acuity (BCVA) better than 0.3 (Snellen chart), central and stable fixation during image acquisition, clear ocular media, and refractive error within ±3.0 diopters spherical or ±2.5 diopters cylindrical. Control participants had no history of significant systemic conditions, including diabetes mellitus, and were not taking any medications at the time of inclusion. Diabetic participants had a confirmed diagnosis of either type I or type II diabetes mellitus at the time of enrollment in the study. They were categorized according to the presence and severity of diabetic retinopathy (no DR, NPDR, or PDR) and had no history of ophthalmic procedures in the three months prior to inclusion.

### 2.3. Ophthalmologic Examination and Imaging Protocol

All participants received a comprehensive ophthalmological examination, which included best-corrected visual acuity testing, refraction, intraocular pressure measurement, and slit-lamp biomicroscopy of both the anterior and posterior segments of the eye. Pharmacologic mydriasis was induced using 10% phenylephrine and 1% tropicamide. Retinal imaging was conducted using a swept-source optical coherence tomography (OCT) device (DRI OCT Triton, Topcon Inc., Tokyo, Japan), complemented by an optical coherence tomography angiography (OCTA) system, from the same manufacturer for en face visualization.

### 2.4. OCTA Image Acquisition and Preprocessing

High-resolution 4.5 × 4.5 mm en face OCTA images (320 × 320 pixels) of the superficial capillary plexus (SCP) were obtained using IMAGEnet 6 software, which automatically segmented the SCP, defined as the layer extending from 2.6 µm below the internal limiting membrane, to 15.6 µm beneath the inner plexiform layer—inner nuclear layer interface.

Images were exported and processed using a custom MATLAB protocol (R2024b, MathWorks, Natick, MA, USA). Each OCTA image of the SCP was processed using five distinct binarization algorithms to analyze vessel density within the parafoveal region (between 1 and 3 mm radius), specifically targeting the four inner subfields of the ETDRS grid, with all analyses performed by the same examiner to ensure consistency. The binarization techniques employed were as follows: local Phansalkar, local Otsu, adaptive mean thresholding, global Otsu thresholding, and global mean thresholding.

**Figure 1:**
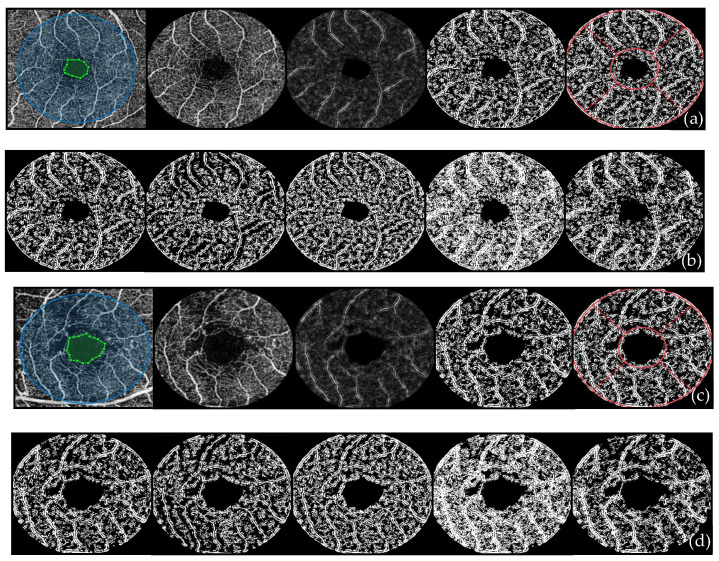
OCTA image processing protocol used to analyze vessel density in the superficial capillary plexus. OCTA images from (**a**,**b**) a healthy volunteer and (**c**,**d**) a patient with diabetic retinopathy. (**a**,**c**): Illustration of the custom-developed MATLAB algorithm. From left to right: first image—manual delineation of the foveal avascular zone (FAZ) (area marked by green dots) and automatic placement of a 3 mm diameter circular region of interest (blue circle); second image: cropped grayscale image with the FAZ excluded; third image: vessel enhancement using a 2D Frangi vesselness filter; forth image: binarized image obtained using the local Phansalkar thresholding method; fifth image: the application of the ETDRS grid on the binarized image. (**b**,**d**): lllustration of binarized OCTA images obtained using five thresholding methods. From left to right: local Phansalkar, local Otsu, local adaptive mean, global mean, and global Otsu.

This study focused on quantifying vessel density in the parafoveal region of the SCP, using a standardized OCTA image processing protocol. The workflow included the following steps:

(1) The foveal avascular zone (FAZ) was manually drawn on each OCTA image, and its centroid was used to define a 3 mm diameter circular region of interest. The resulting region was cropped, and the manually segmented FAZ was excluded from all subsequent analyses.

(2) The cropped image was converted to grayscale for further processing.

(3) Vascular structures were enhanced using a 2D Frangi vesselness filter [19] (applied with a scale range of 1–2, a scale ratio of 2, and sensitivity parameters β_1_ = 0.5 and β_2_ = 15) and the resulting image was inverted to improve vessel-background contrast.

(4) The enhanced image was then binarized using five different thresholding methods: local Phansalkar (15 × 15 window, k = 0.25, *p* = 2, q = 10, R = 1), local Otsu (15 × 15 sliding window, applied to an 8-bit version of the vesselness image), adaptive mean thresholding (15 × 15 window, using MATLAB’s adaptthresh function with sensitivity = 0.5), global mean thresholding (using the average intensity of the entire image), and global Otsu thresholding (using MATLAB’s graythresh function).

(5) After binarization, an ETDRS-like circular grid was applied to the binary image. The parafoveal ring (between 1 and 3 mm radius) was segmented into four quadrants: superior, inferior, nasal, and temporal, with quadrant orientation adjusted for eye laterality.

(6) Vessel density was determined on the binarized images by calculating the ratio of vessel pixels to the total analyzed area. Vessel density was calculated for each parafoveal quadrant (superior, inferior, nasal, and temporal) as well as for the entire parafoveal ring (between 1 and 3 mm radius), defined as the mean of the four sectors (Figure 1).

### 2.5. Statistical Analysis

Statistical analysis was performed using GraphPad Prism (version 10.4.2). Since vascular density was evaluated across two independent factors, a repeated-measures two-way ANOVA was conducted with between-subject factor of group (control, no DR, NPDR, PDR) and within-subject factor of binarization method (five thresholding techniques) to assess the main effects and potential interaction between these factors.

For each binarization method, we then investigated whether a certain quadrant was more likely to be affected with disease progression. For this aim, we used separate repeated-measures two-way ANOVA with between-subject factor of group (control, no DR, NPDR, PDR) and within-subject factor of quadrant (superior, inferior, nasal, temporal).

For all ANOVAs where statistically significant differences were observed for a main factor (*p* < 0.05), we used Tukey’s multiple comparisons test for post hoc pairwise analysis across main factors.

Results from the two-way ANOVAs are reported as F statistics, along with associated degrees of freedom and *p*-values. For multiple comparisons, adjusted *p*-values (*p* adj) from Tukey’s test are presented. All data are expressed as mean ± standard deviation unless otherwise stated.

## 3. Results

### 3.1. Comparison Between Binarization Methods

A two-way repeated-measures ANOVA was performed to evaluate the effects of binarization methods, patient groups, and their interaction on parafoveal vessel density measurements. The analysis revealed a highly significant main effect of binarization method (F(1.776, 115.4) = 1689, *p* < 0.0001). A significant main effect of patient group was also observed (F(3, 65) = 5.773, *p* = 0.0015). Moreover, the interaction between binarization method and patient group reached statistical significance (F(5.328, 115.4) = 3.845, *p* = 0.0024), demonstrating that inter-group differences varied depending on the binarization technique applied.

**Figure 2:**
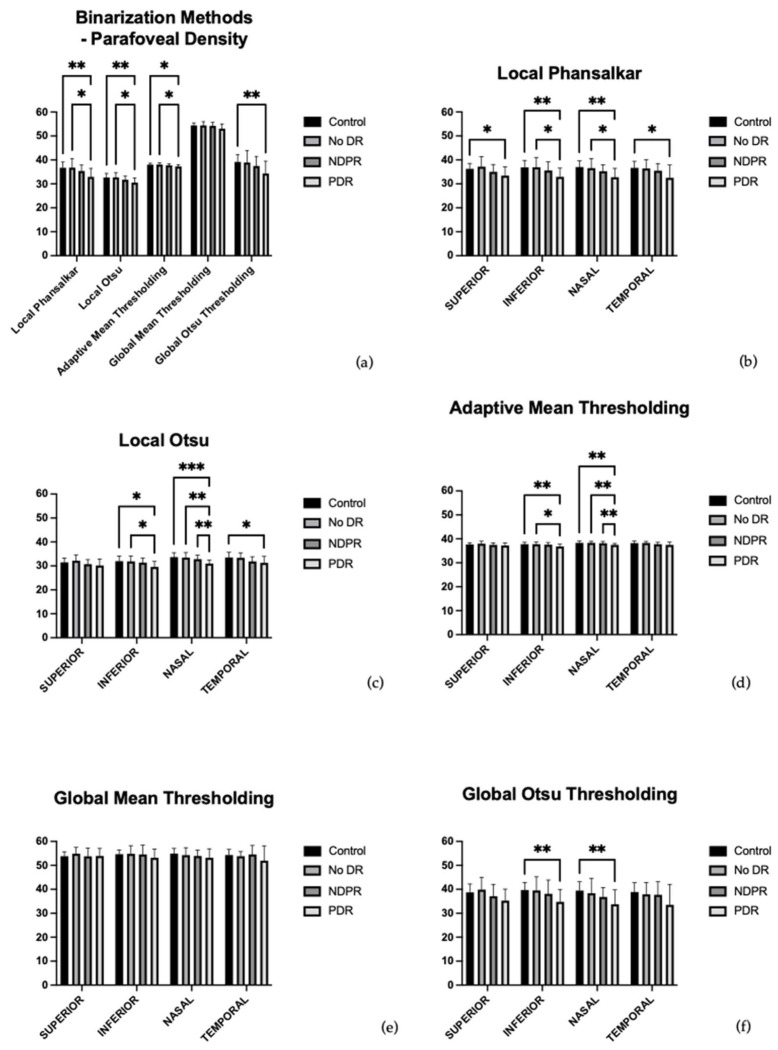
(**a**) Parafoveal vessel density distribution across study groups (control, no DR, NPDR, and PDR) for each binarization method applied (local Phansalkar, local Otsu, adaptive mean thresholding, global mean thresholding, and global Otsu thresholding). (**b**–**f**) Vessel density values in the four quadrants of the inner ETDRS grid (superior, inferior, nasal, and temporal) obtained using (**b**) local Phansalkar, (**c**) local Otsu, (**d**) adaptive mean thresholding, (**e**) global mean thresholding, and (**f**) global Otsu thresholding binarization methods. Statistical significance is indicated as follows: *p* adj < 0.05 (*), *p* adj < 0.01 (**), and *p* adj < 0.001 (***).

Figure 2a presents the average parafoveal vessel density values across four diagnostic groups (control, no DR, NPDR, PDR) and five binarization methods (Figure 2a). Among these, the global mean thresholding consistently produced the highest vessel density values across all groups. Within the local binarization techniques, adaptive mean thresholding yielded the highest density estimates, followed by Phansalkar, while Otsu local consistently produced the lowest values. The global Otsu method yielded intermediate density values, lower than global mean but generally higher than those obtained from local thresholding (Supplementary Table S1).

Post hoc Tukey multiple comparisons revealed a consistent pattern across local binarization methods. Specifically, there was no statistically significant difference between control and NPDR for any of the three local thresholding techniques, indicating limited sensitivity to early microvascular changes. In contrast, statistically significant differences were observed between control and RDP, as well as between no DR and RDP, across all local methods. For local Phansalkar, the adjusted *p*-values were *p* adj (control, RDP) = 0.0029 and *p* adj (no DR, RDP) = 0.0271. Local Otsu yielded *p* adj (control, RDP) = 0.0059 and *p* adj (no DR, RDP) = 0.0176, respectively, while adaptive mean thresholding resulted in *p* adj (control, RDP) = 0.0105 and *p* adj (no DR, RDP) = 0.0118. Although the absolute values differed slightly among methods, this consistent statistical significance highlights the robustness of local approaches in detecting advanced disease.

In contrast to the local methods, the two global binarization strategies demonstrated limited group-level discriminative power. The global mean method, despite producing the highest overall vessel density values, failed to yield any statistically significant differences between patient groups. For example, even the control and RDP comparison did not reach significance *p* adj (control, RDP) = 0.0535, suggesting a tendency to overestimate perfusion and mask pathological differences. The global Otsu method was more conservative, yielding intermediate vessel density values, and detected only one statistically significant comparison between control and RDP *p* adj (control, RDP) = 0.0067. No other pairwise group comparisons reached significance using this method.

### 3.2. Results of Each Binarization Methods

Patient group was defined as a between-subject factor comprising four clinical categories: control, no DR, NPDR, and PDR. The quadrant factor represented a within-subject spatial variable, corresponding to the superior, inferior, nasal, and temporal parafoveal quadrants, in which vessel density was independently quantified. The quadrant × patient group interaction tested whether spatial variations in vessel density differed according to disease stage.

#### 3.2.1. Local Phansalkar

Two-way repeated-measures ANOVA revealed a significant main effect of patient group (F(3, 65) = 6.133, *p* = 0.0010). Neither the quadrant effect (*p* = 0.8267) nor the quadrant × group interaction (*p* = 0.7771) reached statistical significance.

Figure 2b presents the vessel density values in the four quadrants (superior, inferior, nasal, and temporal) of the inner ETDRS grid using the local Phansalkar binarization method (Figure 2b). The corresponding numerical values are provided in Supplementary Table S2.

Post hoc Tukey analysis demonstrated significant reductions in vessel density predominantly in the RDP group, as follows:

- Superior quadrant: *p* adj (control, RDP) = 0.0370,
- Inferior quadrant: *p* adj (control, RDP) = 0.0048, *p* adj (no DR and RDP) = 0.0325,
- Nasal quadrant: *p* adj (control and RDP) = 0.0013, *p* adj (no DR and RDP) = 0.0423,
- Temporal quadrant: *p* adj (control and RDP) = 0.0250.

#### 3.2.2. Local Otsu

The two-way repeated-measures ANOVA demonstrated significant main effects of quadrant (F(2.859, 185.8) = 28.75, *p* < 0.0001) and patient group (F(3, 65) = 5.926, *p* = 0.0012), while the quadrant × group interaction was not significant (*p* = 0.1844).

Figure 2c presents the vessel density values in the four quadrants (superior, inferior, nasal, and temporal) of the inner ETDRS grid using the local Otsu binarization method (Figure 2c). The corresponding numerical values are provided in Supplementary Table S2.

Post hoc Tukey testing identified multiple significant differences involving the RDP group:

- Inferior quadrant: *p* adj (control and RDP) = 0.0119, *p* adj (no DR and RDP) = 0.0388,
- Nasal quadrant: *p* adj (control and RDP) = 0.0003), *p* adj (no DR and RDP) = 0.0048, *p* adj (RDNP and RDP) = 0.0054,
- Temporal quadrant: *p* adj (control and RDP) = 0.0417.

#### 3.2.3. Local Adaptive Mean Thresholding

Repeated-measures ANOVA revealed significant main effects of quadrant (F(2.967, 192.9) = 15.79, *p* < 0.0001) and patient group (F(3, 65) = 5.481, *p* = 0.0020), while the quadrant × group interaction was not significant (*p* = 0.5041).

Figure 2d presents the vessel density values in the four quadrants (superior, inferior, nasal, and temporal) of the inner ETDRS grid using the local adaptive mean binarization method (Figure 2d). The corresponding numerical values are provided in Supplementary Table S2.

Post hoc analysis demonstrated significant vessel density reductions predominantly in advanced disease:

- Inferior quadrant: *p* adj (control and RDP) = 0.0093, *p* adj (no DR and RDP) = 0.0272,
- Nasal quadrant: *p* adj (control and RDP) = 0.0032, *p* adj (no DR and RDP) = 0.0045, *p* adj (RDNP and RDP) = 0.0100.

#### 3.2.4. Global Mean Thresholding

Two-way repeated-measures ANOVA using the global mean binarization method revealed a modest but statistically significant main effect of patient group (F(3, 65) = 3.344, *p* = 0.0244). However, there were no significant effects of quadrant (*p* = 0.7204) or quadrant × group interaction (*p* = 0.8301), indicating that spatial differences across parafoveal quadrants did not vary significantly between groups.

Figure 2e presents the vessel density values in the four quadrants (superior, inferior, nasal, and temporal) of the inner ETDRS grid using the global mean binarization method (Figure 2e). The corresponding numerical values are provided in Supplementary Table S2.

Despite this modest overall group effect, post hoc Tukey analysis failed to identify any statistically significant pairwise differences between patient groups in any individual quadrant.

#### 3.2.5. Global Otsu Thresholding

Repeated-measures ANOVA identified a significant main effect of patient group (F(3, 65) = 4.705, *p* = 0.0049), whereas neither the quadrant effect (*p* = 0.2087) nor the quadrant × group interaction (*p* = 0.8002) was statistically significant.

Figure 2f presents the vessel density values in the four quadrants (superior, inferior, nasal, and temporal) of the inner ETDRS grid using the global Otsu binarization method (Figure 2f). The corresponding numerical values are provided in Supplementary Table S2.

Post hoc analysis revealed limited but consistent inter-group differences, confined to the following:

- Inferior quadrant: *p* adj (control and RDP) = 0.0070,
- Nasal quadrant: *p* adj (control and RDP) = 0.0068.

## 4. Discussion

### 4.1. Two-Dimensional Frangi Filter and Local Versus Global Binarization Methods

The literature showed us that in angiographic imaging, vessel enhancement through filtering is a widely adopted technique used to improve the visibility of vascular structures. Among these methods, the most widely used vesselness filter in the literature is the one introduced by Frangi et al., commonly referred to as the Frangi filter [20,21].

This Hessian-based filter has been successfully applied across various imaging modalities, including magnetic resonance, ultrasound, and photoacoustic angiography [17,22,23,24]. In OCTA, top-hat filtering, optimally oriented flux, Gabor filtering, weighted symmetry filtering, and active shape models [17,25,26]. Despite this variety, the Frangi filter remains a popular and effective choice [17].

The Frangi filter is specifically designed to enhance tubular structures such as blood vessels in medical imaging. It operates by examining the local curvature of the image using the eigenvalues of the Hessian matrix, thereby enhancing areas that exhibit vessel-like characteristics, while suppressing background noise. A central feature of this method is the scale parameter, which allows selective enhancement based on vessel size. By implementing the filter across multiple scales and integrating the responses, the method can simultaneously detect both fine and large vessels, making it highly suitable for analyzing complex vascular structures in OCTA and related imaging modalities [17,21].

However, despite its popularity, the Frangi filter is not without limitations. It has been reported to occasionally miss low signal-to-noise ratio vessels or introduce spurious structures due to background noise. Moreover, the filter’s performance is highly dependent on parameter tuning, especially the range of scale values used, which may limit its robustness across diverse imaging applications. Nevertheless, with careful optimization, the Frangi filter remains a powerful and versatile tool in vessel segmentation and enhancement tasks [17].

Following vessel enhancement with filters like the Frangi filter, a binarization process is required to transform the vesselness map into a binary image, facilitating accurate quantification of vessel density in OCTA images. In essence, thresholding involves classifying pixels as part of the object or background based on whether their intensity values are above or below a defined threshold. The strategy used to determine this threshold can vary substantially and is generally categorized into two main approaches: local and global thresholding.

Local thresholding segments the image into smaller neighborhoods, computing a distinct threshold for each pixel using local intensity statistics, such as the mean and standard deviation of the pixel values within its immediate region. In contrast, global thresholding calculates a single threshold value for the entire image based on the overall intensity histogram, treating the image as a whole [17,18].

Although a wide range of global and local thresholding techniques are available, in our analysis, we evaluated five thresholding methods: three local—local Phansalkar, local Otsu, adaptive mean thresholding, and two global—global mean thresholding, global Otsu thresholding.

Among local adaptive thresholding techniques applied to OCTA image analysis, the Phansalkar method is the most frequently reported, having been employed in numerous studies [21,27,28,29,30,31]. Notably, Chu et al. highlighted the method’s utility in choriocapillaris quantification, while emphasizing the importance of carefully optimizing its parameters to achieve accurate segmentation [21,32]. Other widely used local thresholding approaches include the local mean [21,33] and local median methods [21,31,34]. Additionally, one study implemented a signal-to-noise adaptive binarization technique [21,35], while a few others referenced adaptive thresholding without specifying the exact algorithm used [21,36,37]. Local Otsu thresholding has been widely applied in OCTA image processing to extract vessel attributes and regional properties, by automatically determining an optimal threshold value that separates vessel pixels from the background or non-vessel regions [38].

Global mean thresholding calculates a single threshold value by averaging all pixel intensity values in the image. This threshold is then used to convert the image into binary form by classifying pixels as foreground or background [39,40]. The global Otsu method is a widely used global thresholding algorithm that automatically determines an optimal threshold value by minimizing the intraclass intensity variance between the foreground and background. This technique, commonly applied in the segmentation of OCTA images operates by analyzing the image histogram to identify the threshold that best separates the pixel intensities into two classes, typically represented as black and white pixels, such that the variance within each class is minimized, resulting in a clear binary segmentation [17,18,30].

Based on our findings, the choice of binarization strategy has a significant impact on OCTA derived vessel density measurements within the inner ETDRS grid. Global thresholding methods, particularly global mean, consistently overestimated vessel density, likely due to their non-adaptive nature and the inclusion of background noise. This resulted in reduced sensitivity for detecting microvascular differences between diabetic retinopathy stages.

In contrast, local thresholding methods offered more anatomically consistent and accurate segmentation, better capturing clinically meaningful microvascular changes. These methods demonstrated a greater ability to reveal statistically significant differences across diagnostic groups, reinforcing their value in both clinical and research applications. Overall, our results support the preferential use of local thresholding techniques for more reliable and sensitive OCTA-based quantification in diabetic retinopathy evaluation.

### 4.2. Parafoveal Vessel Density of the SCP

From the literature, it is well established that as diabetic retinopathy progresses, pathological loss of blood vessels occurs throughout the retina [41].

Saif et al. demonstrated a reduction in vessel density at the superficial capillary plexus in diabetic patients. In their analysis, a highly statistically significant decrease in vessel density was observed when the control group was compared with both the NPDR and PDR groups. Furthermore, their results showed a similarly significant reduction in superficial vessel density when comparing the no DR group with the PDR group [42]. In agreement with these findings, Kim et al. reported a progressive decrease in capillary density and branching complexity, accompanied by an increase in average vascular caliber across different stages of diabetic retinopathy. However, they did not identify significant differences in these parameters between healthy subjects and patients with mild NPDR [34,42]. In contrast, Agemy et al. observed a significantly reduced vessel density in the superficial vascular plexus even in mild NPDR compared with control subjects [42,43].

In our previous study, using a local binarization method, we likewise demonstrated a significantly lower SCP vessel density in the PDR group compared with both the control group and the no DR group. However, no statistically significant difference was observed between the control and NPDR groups [44].

In the present study, we further expanded our investigation by applying both local and global binarization thresholding methods to explore their impact on vessel density measurements. Using all three local thresholding approaches (local Phansalkar, local Otsu and adaptive mean thresholding), we consistently observed statistically significant differences between the PDR group and both the control and no DR groups. A similar difference between the PDR and control groups was also detected with one global method, namely global Otsu thresholding. In contrast, no statistically significant differences were observed between the control and NPDR groups, in agreement with our previous findings.

### 4.3. Vessel Density in the Four Quadrants of the Inner ETDRS Grid

Several studies in the literature suggest that a more detailed analysis of the inner ETDRS quadrants may reveal statistically significant differences between groups, either across all quadrants or confined to specific regions.

Saif et al. reported quadrant-specific reductions in superficial vessel density measured at the superficial capillary plexus using 3 × 3 mm OCTA scans. Compared with controls, the no DR group showed significant decreases in the superior, inferior, and nasal quadrants, but not in the temporal quadrant. In contrast, comparisons between controls and both NPDR and PDR groups revealed significant reductions across all quadrants. Similarly, vessel density was significantly lower in all quadrants when comparing the no DR group with the PDR group, while comparisons between NPDR and PDR showed no significant difference in the superior quadrant but significant reductions in the remaining quadrants [42].

In our study, no statistically significant differences in vessel density were observed between the control group and either the no DR or NPDR groups across any quadrant, regardless of the local or global binarization method applied. In contrast, statistically significant differences between the control and PDR groups were consistently detected using all three local thresholding methods and with global Otsu thresholding, predominantly in the nasal and inferior quadrants. In addition, using the local Phansalkar method, significant differences between the control and PDR groups were also observed in the superior and temporal quadrants, while local Otsu identified a significant difference in the temporal quadrant only.

Furthermore, statistically significant differences between no DR and PDR groups were consistently present across all three local binarization methods in the nasal and inferior quadrants. Comparisons between the NPDR and PDR groups revealed statistically significant differences only when using two local thresholding approaches, local Otsu and adaptive mean thresholding, again confined to the nasal quadrant.

Other studies have shown that the nasal sector of the ETDRS grid, as well as the nasal retina, tends to exhibit lower vessel density in patients with diabetic retinopathy.

Chaher et al. demonstrated that only zone 8 of the macular grid, located immediately adjacent to the optic nerve head and corresponding to the nasal outer quadrant of the ETDRS grid (3–6 mm ring), showed lower superficial capillary plexus vessel density in eyes with more severe diabetic retinopathy within the NPDR group [45].

Ma et al. investigated nasal–temporal vascular divergence and suggested a strong association with subsequent diabetic retinopathy progression. They identified two opposing patterns of vascular change. Specifically, in the superficial vascular complex of the diabetic group, vessel density was significantly increased in the temporal quadrant (T6, T11) but decreased in the nasal quadrant (N11, N16, N21) compared with normal controls. The numeric labels correspond to quadrants within concentric annular regions at distances of 3–6 mm (T/N6), 6–11 mm (T/N11), 11–16 mm (T/N16), and 16–21 mm (T/N21) from the foveal center. The authors suggested that this phenomenon may reflect anatomical differences in retinal vasculature, whereby increased superficial vascular complex density corresponds to higher metabolic demand, while reduced density indicates diabetic retinopathy progression. They further proposed that nasal retinal vessels, being closer to the central arterial system, are exposed to higher hemodynamic pressure and are therefore more susceptible to early disease, whereas the larger temporal vessels, with greater blood flow, exhibit stronger compensatory capacity, making the nasal region more vulnerable to progression [41].

In line with these observations, our study demonstrated that the most consistent statistically significant differences, mostly in comparisons involving the PDR group, were observed in the nasal quadrant, followed by the inferior quadrant, despite measurements being restricted to the inner ETDRS ring (1–3 mm) rather than more peripheral concentric parafoveal regions, as reported in the studies mentioned above. This finding may suggest a potential anatomical vulnerability of the nasal area to microvascular compromise in diabetic retinopathy and supports its potential utility as a regional biomarker of disease severity.

In conclusion, local adaptive thresholding methods appear to be the most suitable approaches for parafoveal vessel density analysis in diabetic retinopathy. These methods provide an optimal balance between sensitivity and structural preservation, enabling more accurate detection of disease-related microvascular changes. In contrast, some global methods, such as global mean thresholding, although producing higher numerical vessel density values, lack sufficient discriminative power and may therefore be less reliable for both research and clinical applications where accurate disease-stage stratification is essential. Although the presence and distribution of statistically significant differences across quadrants may vary depending on the specific local binarization method applied, the distinction between the PDR group and both the control and no DR groups remained consistent across all local methods, particularly in the nasal and inferior quadrants.

Taken together, these findings underscore the importance of binarization strategy selection and support the preferential use of local adaptive methods for robust, quadrant-based OCTA assessment in diabetic retinopathy.

### 4.4. Limitations

The foveal avascular zone was manually delineated, and the ETDRS grid was centered based on this manual segmentation. Although this approach may introduce a degree of subjectivity, measurement variability was minimized by having all analyses performed by the same examiner.

A limitation of this study is the inclusion of NPDR and PDR patients with prior retinal treatments. Although all interventions occurred at least three months before enrollment, residual long-term effects on vascular parameters cannot be excluded, and observed differences may reflect both disease severity and treatment history. Previous studies indicate that anti-VEGF therapy can promote partial retinal reperfusion and vascular remodeling [46,47]. As several NPDR and PDR patients had received anti-VEGF treatment prior to imaging, post-treatment measurements may underestimate the true extent of pre-treatment capillary dropout and vascular dilation, partially masking baseline disease severity. Panretinal photocoagulation (PRP) has been shown to modify OCTA-derived retinal microvascular metrics, with studies reporting increased macular vessel density following treatment [48,49]. These changes are thought to result from redistribution of blood flow toward the posterior pole and increased subfoveal choroidal perfusion [50,51,52], potentially leading to an underestimation of baseline ischemic damage when imaging is performed after PRP. Pars plana vitrectomy may improve OCTA-derived macular perfusion metrics in PDR, even in cases where the vitrectomy did not directly influence the macular status [53]. By contrast, other studies demonstated that pars plana vitrectomy may indeed relieve traction, but it does not reliably restore capillary networks, with overall retinal ischemia remaining largely unchanged before and after surgery [54]. This shows that the results of our study would either remain unchanged or underestimate the OCTA metrics analyzed.

## Data Availability

The original contributions presented in the study are included in the article/Supplementary Materials; further inquiries can be directed to the corresponding authors.

## Supplementary Materials

The following supporting information can be downloaded at https://www.mdpi.com/article/10.3390/diagnostics16060934/s1, Table S1: Parafoveal vessel density (mean ± standard deviation) across study groups (control, no DR, NPDR, PDR) according to the binarization method applied (local Phansalkar, local Otsu, adaptive mean thresholding, global mean thresholding, global Otsu thresholding); Table S2. Vessel density values (mean ± standard deviation) in the four quadrants (superior, inferior, nasal, and temporal) of the inner ETDRS grid by study groups (control, no DR, NPDR, PDR) according to the binarization method applied (local Phansalkar, local Otsu, adaptive mean thresholding, global mean thresholding, global Otsu thresholding).

## Abbreviations

DR	diabetic retinopathy
NPDR	non-proliferative diabetic retinopathy
PDR	proliferative diabetic retinopathy
OCTA	optical coherence tomography angiography
SCP	superficial capillary plexus
DCP	deep capillary plexus
FAZ	foveal avascular zone
ETDRS	The Early Treatment Diabetic Retinopathy Study
No DR	diabetic patients without diabetic retinopathy
Anti-VEGF	anti-vascular endothelial growth factor
BCVA	best-corrected visual acuity
OCT	optical coherence tomography
PRP	panretinal photocoagulation

## References

[1] AbdelAl O., Ashraf M., Sampani K., Sun J.K. (2019). “For Mass Eye and Ear Special Issue” Adaptive Optics in the Evaluation of Diabetic Retinopathy. Semin. Ophthalmol..

[2] International Diabetes Federation (2025). Diabetes Atlas.

[3] Wong T.Y., Cheung C.M., Larsen M., Sharma S., Simó R. (2016). Diabetic retinopathy. Nat. Rev. Dis. Primers.

[4] Vaughan M., Denmead P., Tay N., Rajendram R., Michaelides M., Patterson E. (2025). How early can we detect diabetic retinopathy? A narrative review of imaging tools for structural assessment of the retina. Graefe’s Arch. Clin. Exp. Ophthalmol..

[5] Sun Z., Yang D., Tang Z., Ng D.S., Cheung C.Y. (2021). Optical coherence tomography angiography in diabetic retinopathy: An updated review. Eye.

[6] Yu L., Chen Z. (2010). Doppler variance imaging for three-dimensional retina and choroid angiography. J. Biomed. Opt..

[7] Jia Y., Tan O., Tokayer J., Potsaid B., Wang Y., Liu J.J., Kraus M.F., Subhash H., Fujimoto J.G., Hornegger J. (2012). Split-spectrum amplitude-decorrelation angiography with optical coherence tomography. Opt. Express.

[8] Jia Y., Bailey S.T., Hwang T.S., McClintic S.M., Gao S.S., Pennesi M.E., Flaxel C.J., Lauer A.K., Wilson D.J., Hornegger J. (2015). Quantitative optical coherence tomography angiography of vascular abnormalities in the living human eye. Proc. Natl. Acad. Sci. USA.

[9] Nemiroff J., Kuehlewein L., Rahimy E., Tsui I., Doshi R., Gaudric A., Gorin M.B., Sadda S., Sarraf D. (2016). Assessing Deep Retinal Capillary Ischemia in Paracentral Acute Middle Maculopathy by Optical Coherence Tomography Angiography. Am. J. Ophthalmol..

[10] Simonett J.M., Scarinci F., Picconi F., Giorno P., De Geronimo D., Di Renzo A., Varano M., Frontoni S., Parravano M. (2017). Early microvascular retinal changes in optical coherence tomography angiography in patients with type 1 diabetes mellitus. Acta Ophthalmol..

[11] Ishibazawa A., Nagaoka T., Takahashi A., Omae T., Tani T., Sogawa K., Yokota H., Yoshida A. (2015). Optical Coherence Tomography Angiography in Diabetic Retinopathy: A Prospective Pilot Study. Am. J. Ophthalmol..

[12] Yu S., Pang C.E., Gong Y., Freund K.B., Yannuzzi L.A., Rahimy E., Lujan B.J., Tabandeh H., Cooney M.J., Sarraf D. (2015). The spectrum of superficial and deep capillary ischemia in retinal artery occlusion. Am. J. Ophthalmol..

[13] Freiberg F.J., Pfau M., Wons J., Wirth M.A., Becker M.D., Michels S. (2016). Optical coherence tomography angiography of the foveal avascular zone in diabetic retinopathy. Graefe’s Arch. Clin. Exp. Ophthalmol..

[14] Hwang T.S., Jia Y., Gao S.S., Bailey S.T., Lauer A.K., Flaxel C.J., Wilson D.J., Huang D. (2015). Optical coherence tomography angiography features of diabetic retinopathy. Retina.

[15] Hwang T.S., Gao S.S., Liu L., Lauer A.K., Bailey S.T., Flaxel C.J., Wilson D.J., Huang D., Jia Y. (2016). Automated Quantification of Capillary Nonperfusion Using Optical Coherence Tomography Angiography in Diabetic Retinopathy. JAMA Ophthalmol..

[16] Maruko I., Arakawa H., Koizumi H., Izumi R., Sunagawa H., Iida T. (2016). Age-Dependent Morphologic Alterations in the Outer Retinal and Choroidal Thicknesses Using Swept Source Optical Coherence Tomography. PLoS ONE.

[17] Untracht G.R., Matos R.S., Dikaios N., Bapir M., Durrani A.K., Butsabong T., Campagnolo P., Sampson D.D., Heiss C., Sampson D.M. (2021). OCTAVA: An open-source toolbox for quantitative analysis of optical coherence tomography angiography images. PLoS ONE.

[18] Freedman I.G., Li E., Hui L., Adelman R.A., Nwanyanwu K., Wang J.C. (2022). The Impact of Image Processing Algorithms on Optical Coherence Tomography Angiography Metrics and Study Conclusions in Diabetic Retinopathy. Transl. Vis. Sci. Technol..

[19] Untracht G.R., Durkee M.S., Zhao M., Kwok-Cheung Lam A., Sikorski B.L., Sarunic M.V., Andersen P.E., Sampson D.D., Chen F.K., Sampson D.M. (2024). Towards standardising retinal OCT angiography image analysis with open-source toolbox OCTAVA. Sci. Rep..

[20] Frangi A.F., Niessen W.J., Vincken K.L., Viergever M.A. Multiscale vessel enhancement filtering. Proceedings of the Medical Image Computing and Computer-Assisted Intervention—MICCAI’98.

[21] Meiburger K.M., Salvi M., Rotunno G., Drexler W., Liu M. (2021). Automatic Segmentation and Classification Methods Using Optical Coherence Tomography Angiography (OCTA): A Review and Handbook. Appl. Sci..

[22] Chapman B.E., Parker D.L. (2005). 3D multi-scale vessel enhancement filtering based on curvature measurements: Application to time-of-flight MRA. Med. Image Anal..

[23] Hennersperger C., Baust M., Waelkens P., Karamalis A., Ahmadi S.A., Navab N. (2015). Multi-scale tubular structure detection in ultrasound imaging. IEEE Trans. Med. Imaging.

[24] Zhao H., Wang G., Lin R., Gong X., Song L., Li T., Wang W., Zhang K., Qian X., Zhang H. (2018). Three-dimensional Hessian matrix-based quantitative vascular imaging of rat iris with optical-resolution photoacoustic microscopy in vivo. J. Biomed. Opt..

[25] Stefan S., Lee J. (2020). Deep learning toolbox for automated enhancement, segmentation, and graphing of cortical optical coherence tomography microangiograms. Biomed. Opt. Express.

[26] Tan B., Sim R., Chua J., Wong D.W.K., Yao X., Garhöfer G., Schmidl D., Werkmeister R.M., Schmetterer L. (2020). Approaches to quantify optical coherence tomography angiography metrics. Ann. Transl. Med..

[27] Phansalkar N., More S., Sabale A., Joshi M. Adaptive local thresholding for detection of nuclei in diversity stained cytology images. Proceedings of the 2011 International Conference on Communications and Signal Processing.

[28] Laiginhas R., Cabral D., Falcão M. (2020). Evaluation of the different thresholding strategies for quantifying choriocapillaris using optical coherence tomography angiography. Quant. Imaging Med. Surg..

[29] Borrelli E., Sacconi R., Querques L., Battista M., Bandello F., Querques G. (2020). Quantification of diabetic macular ischemia using novel three-dimensional optical coherence tomography angiography metrics. J. Biophotonics.

[30] Mehta N., Liu K., Alibhai A.Y., Gendelman I., Braun P.X., Ishibazawa A., Sorour O., Duker J.S., Waheed N.K. (2019). Impact of binarization thresholding and brightness/contrast adjustment methodology on optical coherence tomography angiography image quantification. Am. J. Ophthalmol..

[31] Su L., Ji Y.-S., Tong N., Sarraf D., He X., Sun X., Xu X., Sadda S.R. (2020). Quantitative assessment of the retinal microvasculature and choriocapillaris in myopic patients using swept-source optical coherence tomography angiography. Graefe’s Arch. Clin. Exp. Ophthalmol..

[32] Chu Z., Cheng Y., Zhang Q., Zhou H., Dai Y., Shi Y., Gregori G., Rosenfeld P.J., Wang R.K. (2020). Quantification of choriocapillaris with phansalkar local thresholding: Pitfalls to avoid. Am. J. Ophthalmol..

[33] Chu Z., Lin J., Gao C., Xin C., Zhang Q., Chen C.-L., Roisman L., Gregori G., Rosenfeld P.J., Wang R.K. (2016). Quantitative assessment of the retinal microvasculature using optical coherence tomography angiography. J. Biomed. Opt..

[34] Kim A.Y., Chu Z., Shahidzadeh A., Wang R.K., Puliafito C.A., Kashani A.H. (2016). Quantifying microvascular density and morphology in diabetic retinopathy using spectral-domain optical coherence tomography angiography. Investig. Ophthalmol. Vis. Sci..

[35] Zhang Y., Li H., Cao T., Chen R., Qiu H., Gu Y., Li P. (2021). Automatic 3D adaptive vessel segmentation based on linear relationship between intensity and complex-decorrelation in optical coherence tomography angiography. Quant. Imaging Med. Surg..

[36] Alam M., Toslak D., Lim J.I., Yao X. (2019). OCT feature analysis guided artery-vein differentiation in OCTA. Biomed. Opt. Express.

[37] Alam M., Le D., Lim J.I., Chan R.V.P., Yao X. (2019). Supervised Machine Learning Based Multi-Task Artificial Intelligence Classification of Retinopathies. J. Clin. Med..

[38] BahadarKhan K., Amir A.K., Shahid M. (2016). A Morphological Hessian Based Approach for Retinal Blood Vessels Segmentation and Denoising Using Region Based Otsu Thresholding. PLoS ONE.

[39] Arrigo A., Aragona E., Saladino A., Amato A., Bandello F., Battaglia Parodi M. (2021). The impact of different thresholds on optical coherence tomography angiography images binarization and quantitative metrics. Sci. Rep..

[40] Glasbey C.A. (1993). An analysis of histogram-based thresholding algorithms. CVGIP Graph. Models Image Process..

[41] Ma Z., Yu F., Li H., Liu Y., Deng B., Zeng Y. (2026). Divergent nasal-temporal changes in superficial vascular density as early biomarkers of diabetic retinopathy. BMC Ophthalmol..

[42] Saif P.S., Salman A.E.G., Omran N.A.H., Farweez Y.A.T. (2020). Assessment of Diabetic Retinopathy Vascular Density Maps. Clin. Ophthalmol..

[43] Agemy S.A., Scripsema N.K., Shah C.M., Chui T., Garcia P.M., Lee J.G., Gentile R.C., Hsiao Y.-S., Zhou Q., Ko T. (2015). Retinal vascular perfusion density mapping using optical coherence tomography angiography in normals and diabetic retinopathy patients. Retina.

[44] Mirescu A.E., Deleanu D.G., Jurja S., Popa-Cherecheanu A., Balta F., Garhofer G., Balta G., Cristescu I.E., Tofolean I.T. (2025). Multimodal Imaging of Diabetic Retinopathy: Insights from Optical Coherence Tomography Angiography and Adaptive Optics. Diagnostics.

[45] Chaher A., Fajnkuchen F., Tabary S., Giocanti-Aurégan A. (2022). Reduced Vessel Density in the Mid-Periphery and Peripapillary Area of the Superficial Capillary Plexus in Non-Proliferative Diabetic Retinopathy. J. Clin. Med..

[46] Kim Y.J., Yeo J.H., Son G., Kang H., Sung Y.S., Lee J.Y., Kim J.-G., Yoon Y.H. (2020). Efficacy of intravitreal AFlibercept injection For Improvement of retinal Nonperfusion In diabeTic retinopathY (AFFINITY study). BMJ Open Diabetes Res. Care.

[47] Massengill M.T., Cubillos S., Sheth N., Sethi A., Lim J.I. (2024). Response of diabetic macular edema to anti-VEGF medications correlates with improvement in macular vessel architecture measured with OCT angiography. Ophthalmol. Sci..

[48] Abdelhalim A.S., Abdelkader M.F.S.O., Mahmoud M.S.E.-D., Mohamed Mohamed A.A. (2022). Macular vessel density before and after panretinal photocoagulation in patients with proliferative diabetic retinopathy. Int. J. Retin. Vitr..

[49] Chatziralli I., Dimitriou E., Agapitou C., Kazantzis D., Kapsis P., Morogiannis N., Kandarakis S., Theodossiadis G., Theodossiadis P. (2023). Optical Coherence Tomography Angiography Changes in Macular Area in Patients with Proliferative Diabetic Retinopathy Treated with Panretinal Photocoagulation. Biomedicines.

[50] Fawzi A.A., Fayed A.E., Linsenmeier R.A., Gao J., Yu F. (2019). Improved macular capillary flow on optical coherence tomography angiography after panretinal photocoagulation for proliferative diabetic retinopathy. Am. J. Ophthalmol..

[51] Takahashi A., Nagaoka T., Sato E., Yoshida A. (2008). Effect of panretinal photocoagulation on choroidal circulation in the foveal region in patients with severe diabetic retinopathy. Br. J. Ophthalmol..

[52] Zhao T., Chen Y., Liu D., Stewart J.M. (2021). Optical coherence tomography angiography assessment of macular choriocapillaris and choroid following panretinal photocoagulation in a diverse population with advanced diabetic retinopathy. Asia-Pac. J. Ophthalmol..

[53] Petrou Sr P., Angelidis C.D., Andreanos K., Kanakis M., Kandarakis S., Karamaounas A., Papakonstantinou E., Mamas N., Droutsas K., Georgalas I. (2021). Reduction of foveal avascular zone after vitrectomy demonstrated by optical coherence tomography angiography. Cureus.

[54] Russell J.F., Scott N.L., Townsend J.H., Shi Y., Gregori G., Crane A.M., Flynn H.W., Sridhar J., Rosenfeld P.J. (2021). Wide-field swept-source optical coherence tomography angiography of diabetic tractional retinal detachments before and after surgical repair. Retina.

