# Supplementary material for: Local Versus Global Binarization Techniques After Frangi Filtering for Optical Coherence Tomography Angiography Based Retinal Vessel Density Assessment in Diabetic Retinopathy
Source: Diagnostics (Basel). 2026 Mar 21;16(6):934. doi: 10.3390/diagnostics16060934 (PMC13025192; doi:10.3390/diagnostics16060934)
Supplement: Supplementary file 1 [file diagnostics-16-00934-s001.zip › diagnostics-4172156-supplementary.pdf]

Table S1. Parafoveal vessel density (mean ± standard deviation) across study groups (control, no DR, NPDR, PDR) according to the binarization method applied (local Phansalkar, local Otsu, adaptive mean thresholding, global mean thresholding, global Otsu thresholding).

Binarization Method	Control (parafoveal density)	No DR (parafoveal density)	NPDR (parafoveal density)	PDR (parafoveal density)
Local Phsansalkar	36,704% ± 2,419%	36,785% ± 3,777%	35,351% ± 2,525%	32,914% ± 3,570%
Local Otsu	32,651% ± 1,718%	32,721% ± 2,018%	31,712% ± 1,495%	30,511% ± 1,947%
Adaptive mean	38,016% ± 0,619%	38,087% ± 0,678%	37,751% ± 0,632%	37,261% ± 0,759%
Global mean	54,432% ± 1,067%	54,450% ± 1,532%	54,208% ± 1,463%	53,054% ± 1,978%
Global Otsu	39,186% ± 3,108%	38,926% ± 4,987%	37,454% ± 4,045%	34,343% ± 5,129%

Table S2. Vessel density values (mean ± standard deviation) in the four quadrants (superior, inferior, nasal, and temporal) of the inner ETDRS grid by study groups (control, no DR, NPDR, PDR) according to the binarization method applied (local Phansalkar, local Otsu, adaptive mean thresholding, global mean thresholding, global Otsu thresholding).

Binarization Method and Quadrant	Control	No DR	NPDR	PDR
Local Phansalkar - Superior	36,251% ± 2,266%	37,181% ± 4,198%	35,022% ± 3,071%	33,448% ± 3,667%
Local Phansalkar - Inferior	36,852% ± 2,922%	36,937% ± 4,089%	35,600% ± 3,583%	32,929% ± 3,655%
Local Phansalkar – Nasal	37,038% ± 2,595%	36,573% ± 4,011%	35,282% ± 2,676%	32,759% ± 3,707%
Local Phansalkar - Temporal	36,678% ± 2,736%	36,447% ± 3,636%	35,496% ± 2,993%	32,522% ± 5,376%

Local Otsu - Superior	31,453% ± 1,822%	32,159% ± 2,467%	30,736% ± 1,893%	30,184% ± 2,673%
Local Otsu - Inferior	31,979% ± 2,137%	31,876% ± 2,324%	31,392% ± 1,945%	29,589% ± 2,284%
Local Otsu - Nasal	33,664% ± 1,875%	33,474% ± 2,092%	32,826% ± 1,658%	30,998% ± 1,457%
Local Otsu - Temporal	33,506% ± 2,170%	33,375% ± 2,100%	31,891% ± 1,911%	31,276% ± 2,713%
Adaptive mean - Superior	37,686% ± 0,684%	37,976% ± 1,053%	37,467% ± 0,835%	37,237% ± 1,038%
Adaptive mean - Inferior	37,796% ± 0,803%	37,783% ± 0,893%	37,559% ± 0,901%	36,841% ± 0,916%
Adaptive mean – Nasal	38,328% ± 0,746%	38,303% ± 0,663%	38,192% ± 0,717%	37,477% ± 0,574%
Adaptive mean - Temporal	38,249% ± 0,848%	38,291% ± 0,634%	37,789% ± 0,781%	37,494% ± 1,117%
Global mean - Superior	53,814% ± 1,857%	54,874% ± 2,629%	53,760% ± 3,466%	53,931% ± 3,149%
Global mean - Inferior	54,671% ± 1,818%	54,844% ± 3,375%	54,572% ± 3,910%	53,173% ± 3,598%
Global mean - Nasal	54,902% ± 2,157%	54,277% ± 3,095%	53,932% ± 2,427%	53,154% ± 3,786%
Global mean - Temporal	54,346% ± 2,341%	53,805% ± 1,976%	54,567% ± 3,758%	51,964% ± 6,188%
Global Otsu - Superior	38,719% ± 3,494%	39,888% ± 5,140%	37,137% ± 4,953%	35,280% ± 4,792%
Global Otsu - Inferior	39,701% ± 3,197%	39,531% ± 5,708%	38,148% ± 5,752%	34,789% ± 5,205%

Global Otsu - Nasal	39,469% ± 3,724%	38,380% ± 6,227%	36,809% ± 3,957%	33,784% ± 5,985%
Global Otsu - Temporal	38,856% ± 3,939%	37,906% ± 4,926%	37,721% ± 5,458%	33,523% ± 8,571%
